# Reliability of B-mode ultrasound measurement of carotid intima-media thickness in assessing cardiovascular risk: a cross-sectional study

**DOI:** 10.3389/fcvm.2026.1740930

**Published:** 2026-01-30

**Authors:** Ru Zhao, Changhe Yuan, Guiping Zhang, Fang Ma

**Affiliations:** Department of Ultrasound, The Second People’s Hospital of Hefei, Hefei Hospital Affiliated to Anhui Medical University, Hefei, Anhui, China

**Keywords:** atherosclerosis, B-mode ultrasound, cardiovascular risk, carotid intima-media thickness, framingham risk score

## Abstract

**Objective:**

Carotid intima-media thickness (CIMT) is considered a marker of subclinical atherosclerosis; however, its reliability in predicting cardiovascular risk across different populations remains controversial. This study aimed to evaluate the correlation between B-mode ultrasound-measured CIMT and traditional cardiovascular risk scoring systems, and to investigate its value as a marker of cardiovascular risk.

**Methods:**

This cross-sectional study included 328 asymptomatic adults (excluding those with established cardiovascular disease). High-resolution B-mode ultrasound was used to measure CIMT bilaterally in the common carotid artery (CCA), carotid bulb, and internal carotid artery (ICA), and to calculate mean and maximum CIMT values. The Framingham Risk Score (FRS) and Pooled Cohort Equations (PCE) were computed, and data on traditional cardiovascular risk factors were collected. Pearson or Spearman correlation analyses, ROC curve analysis, and multivariate regression analysis were used to assess the relationship between CIMT and cardiovascular risk.

**Results:**

The mean age of the 328 participants was 59.3 ± 10.2 years, with males comprising 48.2% of the cohort. The mean CIMT was 0.74 ± 0.09 mm, and the maximum CIMT was 0.95 ± 0.11 mm. CIMT showed significant positive correlations with both FRS (*r* = 0.68, *p* < 0.001) and PCE (*r* = 0.64, *p* < 0.001).Multivariate analysis demonstrated that mean CIMT (OR = 1.46, 95% CI: 1.23–1.72) and maximum CIMT (OR = 1.58, 95% CI: 1.31–1.91) were independently associated with high cardiovascular risk categories as defined by FRS and PCE after adjusting for traditional risk factors. ROC curve analysis revealed that maximum CIMT had an AUC of 0.79 (95% CI: 0.73–0.85) for identifying high FRS risk (>20%) and an AUC of 0.76 (95% CI: 0.70–0.82) for identifying high PCE risk (>7.5%).

**Conclusion:**

B-mode ultrasound-measured CIMT significantly correlates with traditional cardiovascular risk scores and is independently associated with high cardiovascular risk categories after adjustment for traditional risk factors. CIMT measurements of the carotid bulb appear to show stronger correlation with risk scores than common carotid artery measurements. CIMT demonstrates correlation with established risk scoring systems and shows the most significant risk reclassification effect in intermediate-risk populations, supporting its potential utility as a complementary assessment tool.

## 1 Introduction

Cardiovascular disease (CVD) remains one of the leading causes of death globally, with approximately 17.9 million deaths annually according to the World Health Organization. Early identification of high-risk individuals and implementation of preventive measures are crucial for reducing CVD related mortality (1). Although traditional risk scoring systems such as the Framingham Risk Score (FRS) and the Pooled Cohort Equations for Atherosclerotic Cardiovascular Disease (PCE) are widely used, their predictive accuracy has limitations (2, 3). Consequently, the search for more precise subclinical atherosclerosis indicators has become a focus of current research.

Carotid intima-media thickness (CIMT) has received extensive attention over recent decades as a non-invasive method for assessing subclinical atherosclerosis (4). B-mode ultrasound, the primary tool for measuring CIMT, offers advantages of safety, convenience, and high reproducibility (5). Since Pignoli et al. first reported using ultrasound to measure CIMT in the 1980s (6), numerous studies have demonstrated that increased CIMT is significantly associated with elevated cardiovascular event risk (7, 8). The 2010 American Heart Association (AHA) guidelines indicated that CIMT could serve as a complement to traditional risk assessment, particularly for risk restratification in intermediate-risk populations (9).

However, controversy exists regarding the reliability of CIMT for predicting cardiovascular risk. The IMPROVE study found that the overall mean carotid CIMT had weaker predictive capability for cardiovascular events compared to carotid plaques (10). In contrast, the Rotterdam study showed that for each 0.1 mm increase in CIMT, myocardial infarction risk increased by 10%–15% (11). The MESA study further confirmed that CIMT provides additional predictive information even after adjusting for traditional risk factors (12). These inconsistent results may be related to measurement methods, anatomical site selection, follow-up duration, and study population characteristics (13). Standardization of CIMT measurement is a key factor affecting its predictive reliability. Touboul et al. proposed a consensus statement on CIMT measurement, emphasizing the importance of standardized measurement protocols (14). Stein et al. demonstrated that CIMT from different vascular segments (common carotid artery, carotid bulb, and internal carotid artery) have varying predictive values (15). Additionally, unilateral vs. bilateral measurements and the choice between mean vs. maximum values may also influence predictive outcomes (16).

Demographic characteristics such as age, gender, and ethnicity also influence the relationship between CIMT and cardiovascular risk. A meta-analysis conducted by Lorenz et al. demonstrated that increased CIMT has higher predictive value in older populations (17). Polak et al. found that the ability of CIMT to predict cardiovascular events varies across different ethnic groups (18). Additionally, patients with comorbid risk factors such as hypertension, diabetes, and dyslipidemia show different CIMT progression rates and predictive values (19). A key research focus has been whether CIMT provides incremental predictive value beyond traditional risk scores. A meta-analysis by Den Ruijter et al. indicated that adding CIMT to the FRS model only marginally improved risk prediction accuracy (20). However, Nambi et al. demonstrated that combining CIMT with carotid plaque information significantly enhanced risk prediction capability (21). This suggests that CIMT measurement alone may be insufficient for comprehensive atherosclerotic burden assessment and should be integrated with other indicators. Recent advances in ultrasound technology, such as three-dimensional ultrasound and vascular elastography, offer new directions for improving predictive accuracy in CIMT measurement (22). The concept of “vascular age” proposed by Sillesen et al., which comprehensively evaluates CIMT, plaque burden, and vascular stiffness, may provide a more thorough risk assessment than any single indicator (23).

Given the inconsistency of current research findings and methodological differences, this study aims to evaluate the correlation between B-mode ultrasound CIMT measurement and established cardiovascular risk scores through a cross-sectional design. We will analyze the correlation between CIMT measurements at different anatomical locations and traditional risk scores, assess their predictive value across different risk populations, and explore optimal measurement protocols. Furthermore, this study will examine the value of combining CIMT with other ultrasound indicators (such as plaque presence) to improve predictive accuracy. Through these analyses, we hope to provide more solid evidence for the rational application of CIMT in clinical practice.

## 2 Methods

### 2.1 Study population and sample size calculation

This cross-sectional study was conducted at the Cardiovascular Medicine Outpatient Clinic of the Second People's Hospital of Hefei (Hefei Hospital Affiliated to Anhui Medical University) from January 2020 to June 2025.The sample size was calculated based on the expected correlation coefficient between CIMT and established cardiovascular risk scoring systems (FRS and PCE).Referencing previous studies, particularly the MESA study which reported a correlation coefficient of *r* = 0.64 between CIMT and cardiovascular events, we calculated that a minimum sample size of 298 participants would be required to achieve 80% statistical power with a two-sided α level of 0.05. Accounting for potential dropouts and incomplete data, we aimed to recruit 330 participants. Using consecutive sampling, we recruited 328 participants aged 40–75 years who attended the cardiovascular clinic for routine health assessment or cardiovascular risk evaluation. Inclusion criteria: (1) Adults aged 40–75 years; (2) Presenting to the cardiovascular medicine outpatient clinic for routine health assessment or cardiovascular risk evaluation; (3) Asymptomatic for established cardiovascular disease (see exclusion criteria for details); (4) Willing to undergo carotid ultrasound examination;(5) Able to provide complete medical history and laboratory test data; (6) Voluntarily agreed to participate and provided written informed consent. Exclusion criteria: (1) previous diagnosis of coronary heart disease, stroke, or peripheral arterial disease; (2) recent (<6 months) acute infection or autoimmune disease; (3) malignancy; (4) severe hepatic or renal dysfunction; (5) history of neck surgery; and (6) inadequate visualization of carotid arteries on B-mode ultrasound. All participants provided written informed consent prior to enrollment. This study was approved by the hospital's ethics committee.

### 2.2 Clinical and laboratory assessments

Demographic and clinical data were collected from all participants, including age, sex, smoking history (current or former smoking defined as “yes”), and family history (defined as premature cardiovascular disease in first-degree relatives: males <55 years, females <65 years). Height and weight were measured to calculate body mass index (BMI = weight/height^2^, kg/m^2^). Waist circumference was measured in centimeters.Blood pressure was measured using a standard mercury sphygmomanometer after participants had rested in a seated position for 5 min, with the average of two measurements recorded. Hypertension was defined as systolic blood pressure ≥140 mmHg and/or diastolic blood pressure ≥90 mmHg, or current use of antihypertensive medications.

Venous blood samples were collected from all participants after a 12 h fast to determine total cholesterol (TC), low-density lipoprotein cholesterol (LDL-C), high-density lipoprotein cholesterol (HDL-C), triglycerides (TG), fasting glucose (FG), and glycated hemoglobin (HbA1c). Diabetes was defined as fasting glucose ≥126 mg/dL, HbA1c ≥6.5%, or current use of glucose-lowering medications. Estimated glomerular filtration rate (eGFR) was calculated using the CKD-EPI formula. Information regarding participants' medication use, including lipid-lowering agents, antihypertensive drugs, and antiplatelet medications, was documented.

### 2.3 Cardiovascular risk assessment

The 10-year risk of coronary heart disease was calculated using the FRS(2), while the 10-year risk of atherosclerotic cardiovascular disease was assessed using the PCE(3). Based on FRS results, participants were categorized as low risk (<10%), intermediate risk (10%–20%), or high risk (>20%). Similarly, PCE results were used to classify participants as low risk (<5%), intermediate risk (5%–7.5%), or high risk (>7.5%).

### 2.4 Carotid ultrasonography

Carotid ultrasound examinations were performed by two professionally trained sonographers who were blinded to participants' clinical data. Examinations were conducted using a high-resolution color Doppler ultrasound system (Philips EPIQ 7, Netherlands) equipped with a 7.5–10 MHz linear transducer. Participants were positioned supine with the head slightly tilted backward and rotated approximately 45 degrees to the contralateral side. CIMT was measured bilaterally at the CCA, carotid bulb, and ICA. Three measurements were taken at each site and averaged.

The CCA CIMT was measured at the far wall approximately 1 cm proximal to the carotid bulb along a straight arterial segment. The carotid bulb CIMT was measured at the point where the CCA began to dilate. The ICA CIMT was measured 1 cm distal to the carotid bifurcation. CIMT was defined as the distance from the lumen-intima interface to the media-adventitia interface. The mean CIMT was calculated as the average of all six measurement sites, while the maximum CIMT was defined as the highest value among all measurements. Carotid plaque was defined as a focal lesion with CIMT ≥1.5 mm or a focal thickening ≥50% relative to the surrounding vessel wall. Inter-observer reliability between the two sonographers was assessed using the ICC, which was 0.92, indicating excellent reproducibility of measurements.

### 2.5 Statistical analysis

Statistical analyses were performed using SPSS 25.0 (IBM Corp., Armonk, NY, USA). Continuous variables were presented as mean ± standard deviation for normally distributed data or median and interquartile range for non-normally distributed data; categorical variables were expressed as frequencies and percentages. Student's *t*-test or Mann–Whitney *U* test was used to compare continuous variables, while chi-square test was used for categorical variables.

Pearson or Spearman correlation analyses (depending on data distribution) were conducted to assess the relationship between CIMT and both FRS and PCE scores. Multivariate linear regression analysis was performed to evaluate the association between CIMT and traditional cardiovascular risk factors, as well as its independent correlation with FRS and PCE. Receiver operating characteristic (ROC) curve analysis was used to assess the diagnostic performance of different CIMT measurements (mean CIMT, maximum CIMT, and CIMT at different sites) for predicting high cardiovascular risk. Multivariate logistic regression analysis was employed to evaluate the independent association between CIMT and high cardiovascular risk categories (as defined by FRS and PCE) after adjusting for traditional risk factors. All statistical tests were two-sided, with *P* < 0.05 considered statistically significant.

## 3 Results

### 3.1 Baseline characteristics of study population

A total of 328 participants completed the study, comprising 158 males (48.2%) and 170 females (51.8%), with a mean age of 59.3 ± 10.2 years. The baseline clinical characteristics of the participants are presented in Table 1. The prevalence of hypertension was 46.3% (152/328), diabetes mellitus was 23.8% (78/328), and the smoking rate was 30.5% (100/328). Among participants, 45.7% (150/328) were taking lipid-lowering medications, 44.5% (146/328) were on antihypertensive medications, and 18.0% (59/328) were using antiplatelet agents. Regarding risk stratification, according to the FRS, 30.2% (99/328) of participants were classified as low risk, 42.1% (138/328) as intermediate risk, and 27.7% (91/328) as high risk. Based on the PCE, 33.8% (111/328) were categorized as low risk, 35.1% (115/328) as intermediate risk, and 31.1% (102/328) as high risk. The detection rate of carotid plaques was 37.2% (122/328).

**Table 1:** Baseline characteristics of the study population.

Variables	Total (*n* = 328)	Male(*n* = 158)	Female(*n* = 170)	*P*
Age, year	59.3 ± 10.2	60.1 ± 9.8	58.6 ± 10.5	0.162
BMI, kg/m^2^	26.5 ± 1.8	26.8 ± 1.7	26.2 ± 1.9	0.003
WC, cm	91.7 ± 7.2	96.9 ± 3.9	86.9 ± 6.2	<0.001
SBP, mmHg	137.5 ± 10.8	138.9 ± 10.4	136.2 ± 11.0	0.024
DBP, mmHg	81.1 ± 4.8	83.2 ± 4.6	79.1 ± 4.2	<0.001
Hypertension, *n*(%)	152 (46.3)	83 (52.5)	69 (40.6)	0.031
Diabetes, *n*(%)	78 (23.8)	41 (25.9)	37 (21.8)	0.374
Smoking, *n*(%)	100 (30.5)	76 (48.1)	24 (14.1)	<0.001
Family history, *n*(%)	76 (23.2)	35 (22.2)	41 (24.1)	0.675
Total cholesterol, mg/dL	196.2 ± 10.2	190.4 ± 6.2	201.6 ± 10.8	<0.001
LDL-C, mg/dL	124.6 ± 7.1	122.8 ± 6.6	126.3 ± 7.2	<0.001
HDL-C, mg/dL	49.6 ± 7.3	44.0 ± 2.7	54.9 ± 6.9	<0.001
Triglyceride, mg/dL	134.3 ± 24.3	147.9 ± 17.8	121.5 ± 21.8	<0.001
Glucose, mg/dL	101.4 ± 18.6	104.3 ± 20.4	98.7 ± 16.3	0.007
HbA1c, %	5.8 ± 0.6	5.9 ± 0.6	5.7 ± 0.5	0.002
eGFR, mL/min/1.73 m^2^	83.7 ± 8.2	81.4 ± 9.0	85.9 ± 6.8	<0.001
Lipid-lowering drugs, *n*(%)	150 (45.7)	81 (51.3)	69 (40.6)	0.052
Antihypertensive drugs, *n*(%)	146 (44.5)	79 (50.0)	67 (39.4)	0.053
Antiplatelet therapy, *n*(%)	59 (18.0)	39 (24.7)	20 (11.8)	0.002
FRS,%	14.3 ± 8.2	18.6 ± 7.8	10.3 ± 6.5	<0.001
PCE,%	11.9 ± 8.4	14.7 ± 8.0	9.2 ± 8.0	<0.001
Carotid plaque, *n*(%)	122 (37.2)	72 (45.6)	50 (29.4)	0.002

WC, waist circumference; SBP, systolic blood pressure; DBP, diastolic blood pressure; BMI: body mass index; LDL-C, low-density lipoprotein cholesterol; HDL-C, high-density lipoprotein cholesterol; HbA1c, glycosylated hemoglobin; eGFR, estimated glomerular filtration rate; FRS, framingham risk score; PCE, pooled cohort equations score.

### 3.2 Carotid intima-media thickness measurements

Table 2 presents the CIMT measurements at different carotid artery sites. The carotid bulb demonstrated the highest CIMT values, followed by the common carotid artery and internal carotid artery. The mean CIMT was 0.74 ± 0.09 mm, while the maximum CIMT was 0.95 ± 0.11 mm. Males exhibited significantly higher CIMT values than females at all measurement sites (*P* < 0.001). Participants with carotid plaques had significantly higher CIMT values at all sites compared to those without plaques (*P* < 0.001).

**Table 2:** Carotid intima-media thickness measurements (mm).

CIMT Measure	Total(*n* = 328)	Male(*n* = 158)	Female(*n* = 170)	*P*	Plaque present (*n* = 122)	Non-plaque(*n* = 206)	*P*
Right-CCA	0.70 ± 0.09	0.74 ± 0.08	0.66 ± 0.06	<0.001	0.79 ± 0.07	0.64 ± 0.06	<0.001
Left-CCA	0.69 ± 0.09	0.73 ± 0.08	0.65 ± 0.06	<0.001	0.78 ± 0.07	0.64 ± 0.05	<0.001
Right-Bulb	0.81 ± 0.10	0.85 ± 0.08	0.77 ± 0.08	<0.001	0.91 ± 0.06	0.75 ± 0.06	<0.001
Left-Bulb	0.83 ± 0.10	0.87 ± 0.08	0.79 ± 0.08	<0.001	0.93 ± 0.06	0.77 ± 0.06	<0.001
Right-ICA	0.68 ± 0.07	0.71 ± 0.06	0.65 ± 0.06	<0.001	0.75 ± 0.05	0.63 ± 0.05	<0.001
Left-ICA	0.70 ± 0.07	0.73 ± 0.07	0.67 ± 0.06	<0.001	0.77 ± 0.05	0.65 ± 0.05	<0.001
Mean-CIMT	0.74 ± 0.09	0.77 ± 0.08	0.70 ± 0.07	<0.001	0.82 ± 0.05	0.68 ± 0.05	<0.001
Max-CIMT	0.95 ± 0.11	0.99 ± 0.09	0.91 ± 0.10	<0.001	1.04 ± 0.09	0.89 ± 0.07	<0.001

CCA, common carotid artery; Bulb, carotid bulb; ICA, internal carotid artery; CIMT, carotid intima-media thickness.

### 3.3 Association of CIMT with cardiovascular risk score

CIMT showed significant positive correlations with both FRS and PCE (Table 3). The correlation coefficient between mean CIMT and FRS was *r* = 0.68 (*P* < 0.001), and between mean CIMT and PCE was r = 0.64 (*P* < 0.001). Similarly, maximum CIMT demonstrated significant correlations with FRS (*r* = 0.66, *P* < 0.001) and PCE (*r* = 0.63, *P* < 0.001). Among all measurement sites, CIMT at the carotid bulb exhibited the strongest correlation with cardiovascular risk scores, followed by the common carotid artery and the internal carotid artery.

**Table 3:** Association of CIMT with cardiovascular risk score.

CIMT Measure	FRS		PCE	
	*R*	*P*	*R*	*P*
Right-CCA	0.64	<0.001	0.61	<0.001
Left-CCA	0.63	<0.001	0.60	<0.001
Right-Bulb	0.67	<0.001	0.63	<0.001
Left-Bulb	0.68	<0.001	0.64	<0.001
Right-ICA	0.62	<0.001	0.58	<0.001
Left-ICA	0.63	<0.001	0.59	<0.001
Mean-CIMT	0.68	<0.001	0.64	<0.001
Max-CIMT	0.66	<0.001	0.63	<0.001

FRS, framingham risk score; PCE, pooled cohort equations score; CCA, common carotid artery; Bulb, carotid bulb; ICA, internal carotid artery; CIMT, carotid intima-media thickness.

### 3.4 Association of CIMT with traditional cardiovascular risk factors

Multivariate linear regression analysis revealed that age, male gender, hypertension, diabetes, and smoking were significantly positively associated with both mean and maximum CIMT values (*P* < 0.01), while HDL-C showed a significant negative correlation with CIMT (*P* < 0.01) (Table 4). Among these factors, age (standardized *β* = 0.43, *P* < 0.001) and hypertension (standardized *β* = 0.25, *P* < 0.001) emerged as the strongest independent correlates of CIMT.

**Table 4:** Multiple linear regression analysis: association of CIMT with traditional cardiovascular risk factors.

Variables	Mean-CIMT			Max-CIMT		
	*Β*	Normalized Beta	*P*	*Β*	Normalized Beta	*P*
Age (per additional 10 years)	0.038	0.43	<0.001	0.043	0.41	<0.001
Gender (Male)	0.021	0.12	0.010	0.026	0.12	0.011
BMI (per 1 kg/m ^2^ increase)	0.003	0.06	0.253	0.004	0.07	0.189
WC (per 10 cm increase)	0.008	0.07	0.173	0.010	0.07	0.167
Hypertension (Yes)	0.044	0.25	<0.001	0.049	0.23	<0.001
Diabetes (Yes)	0.025	0.12	0.007	0.028	0.12	0.010
Smoking (Yes)	0.018	0.09	0.036	0.022	0.10	0.032
Family history (Yes)	0.009	0.04	0.327	0.011	0.04	0.324
Total cholesterol (per 10 mg/dL increase)	0.003	0.04	0.442	0.004	0.04	0.436
LDL-C (per 10 mg/dL increase)	0.006	0.05	0.260	0.008	0.05	0.243
HDL-C (per 10 mg/dL increase)	−0.018	−0.14	0.003	−0.021	−0.14	0.004
Triglyceride (per 10 mg/dL increase)	0.002	0.05	0.268	0.002	0.05	0.304
HbA1c (per 1% increase)	0.011	0.07	0.123	0.013	0.07	0.125

WC, waist circumference; BMI, body mass index; LDL-C, low-density lipoprotein cholesterol; HDL-C, high-density lipoprotein cholesterol; HbA1c, glycosylated hemoglobin.

#### 3.4.1 Association of CIMT with novel lipid ratio parameters

To further explore the relationship between lipid metabolism and CIMT, particularly given the absence of significant correlation between LDL-C alone and CIMT, we calculated novel lipid ratio parameters that integrate both atherogenic and anti-atherogenic lipoproteins. These included non-HDL-C/HDL-C ratio, LDL-C/HDL-C ratio, and total cholesterol/HDL-C ratio. Table 4A presents the correlation and regression analyses between these novel lipid parameters and CIMT. All three lipid ratio parameters demonstrated significant positive correlations with both mean and maximum CIMT (all *P* < 0.001). The correlation coefficients ranged from *r* = 0.26 to *r* = 0.28 for mean CIMT and *r* = 0.25 to *r* = 0.27 for maximum CIMT. Multivariate linear regression analysis, after adjusting for age, gender, BMI, hypertension, diabetes, and smoking, revealed that all three lipid ratio parameters remained independently associated with CIMT (Table 4A). Non-HDL-C/HDL-C ratio showed a standardized *β* coefficient of 0.12 (*P* = 0.008), LDL-C/HDL-C ratio showed *β* = 0.11 (*P* = 0.015), and total cholesterol/HDL-C ratio showed *β* = 0.11 (*P* = 0.012) for mean CIMT. These associations remained significant even after adjustment for traditional cardiovascular risk factors, indicating that these lipid ratio parameters provide additional information about atherosclerotic burden beyond what is captured by individual lipid components.

**Table 4A:** Correlation and multiple linear regression analysis: association of CIMT with novel lipid ratio parameters.

Lipid Ratio Parameters	Mean-CIMT	Max-CIMT
Pearson Correlation Analysis		
Non-HDL-C/HDL-C ratio	*r* = 0.28, *P* < 0.001	*r* = 0.27, *P* < 0.001
LDL-C/HDL-C ratio	*r* = 0.26, *P* < 0.001	*r* = 0.25, *P* < 0.001
Total cholesterol/HDL-C ratio	*r* = 0.27, *P* < 0.001	*r* = 0.26, *P* < 0.001
Multivariate Linear Regression Analysis*		
Non-HDL-C/HDL-C ratio	*β* = 0.012, Std *β* = 0.12, *P* = 0.008	*β* = 0.014, Std *β* = 0.11, *P* = 0.011
LDL-C/HDL-C ratio	*β* = 0.015, Std *β* = 0.11, *P* = 0.015	*β* = 0.017, Std *β* = 0.10, *P* = 0.020
Total cholesterol/HDL-C ratio	*β* = 0.009, Std *β* = 0.11, *P* = 0.012	*β* = 0.011, Std *β* = 0.10, *P* = 0.018

Non-HDL-C, total cholesterol—HDL-C; Std *β*, standardized beta coefficient.

* Adjusted for age, gender, BMI, hypertension, diabetes, and smoking.

### 3.5 Diagnostic performance of CIMT in identifying high cardiovascular risk categories

Table 5 presents the results of ROC curve analysis for CIMT in identifying individuals categorized as high FRS risk (>20%) and high PCE risk (>7.5%).The maximum CIMT demonstrated an area under the curve (AUC) of 0.79 (95% CI: 0.73–0.85) for predicting high FRS risk, with an optimal cutoff value of 0.93 mm (sensitivity 72.5%, specificity 74.1%). For predicting high PCE risk, the maximum CIMT showed an AUC of 0.76 (95% CI: 0.70–0.82), with an optimal cutoff value of 0.91 mm (sensitivity 70.6%, specificity 72.1%). Among all measurement sites, the carotid bulb CIMT exhibited the strongest predictive capability, followed by mean CIMT and common carotid artery CIMT.

**Table 5:** ROC curve analysis of CIMT for identifying high cardiovascular risk categories.

CIMTMeasure	FRS High risk(>20%)					PCE High risk(>7.5%)				
	AUC	95% CI	Best Cutoff	Sensitivity (%)	Specificity (%)	AUC	95% CI	Best Cutoff	Sensitivity (%)	Specificity (%)
Right-CCA	0.74	0.68–0.80	0.75	68.1	69.6	0.72	0.66–0.78	0.73	67.6	68.1
Left-CCA	0.73	0.67–0.79	0.74	67.0	68.9	0.71	0.65–0.77	0.72	66.7	67.3
Right-Bulb	0.77	0.71–0.83	0.86	71.4	72.5	0.74	0.68–0.80	0.84	69.6	70.4
Left-Bulb	0.78	0.72–0.84	0.88	72.5	73.2	0.75	0.69–0.81	0.86	70.6	71.2
Right-ICA	0.72	0.66–0.78	0.71	67.0	67.5	0.70	0.64–0.76	0.69	65.7	65.9
Left-ICA	0.73	0.67–0.79	0.73	68.1	68.1	0.71	0.65–0.77	0.71	66.7	66.4
Mean-CIMT	0.78	0.72–0.84	0.79	71.4	73.2	0.75	0.69–0.81	0.77	69.6	70.8
Max-CIMT	0.79	0.73–0.85	0.93	72.5	74.1	0.76	0.70–0.82	0.91	70.6	72.1

FRS, framingham risk score; PCE, pooled cohort equations score; CCA, common carotid artery; Bulb, carotid bulb; ICA, internal carotid artery; CIMT, carotid intima-media thickness; AUC, area under the curve.

### 3.6 Independent association between CIMT and high cardiovascular risk categories

Multivariate logistic regression analysis demonstrated that, after adjusting for age, gender, BMI, hypertension, diabetes, smoking status, HDL-C, and LDL-C, both mean CIMT and maximum CIMT remained independently associated with high FRS risk categories (>20%) and high PCE risk categories (>7.5%) (Table 6). This indicates that CIMT provides additional information beyond traditional risk factors for identifying individuals in high-risk categories as defined by these established scoring systems. For FRS high risk, each 0.1 mm increase in mean CIMT was associated with an adjusted odds ratio (OR) of 1.46 (95% CI: 1.23–1.72, *P* < 0.001), while each 0.1 mm increase in maximum CIMT corresponded to an adjusted OR of 1.58 (95% CI: 1.31–1.91, *P* < 0.001). Similarly, for PCE high risk, each 0.1 mm increase in mean CIMT yielded an adjusted OR of 1.39 (95% CI: 1.18–1.64, *P* < 0.001), and each 0.1 mm increase in maximum CIMT resulted in an adjusted OR of 1.49 (95% CI: 1.24–1.80, *P* < 0.001).

**Table 6:** Multiple logistic regression analysis: independent association between CIMT and high cardiovascular risk categories.

Variables	FRS High risk(>20%)		PCE High risk(>7.5%)	
	Adjusted OR (95% CI)	*P*	Adjusted OR (95% CI)	*P*
Mean-CIMT (per 0.1 mm increment)	1.46 (1.23–1.72)	<0.001	1.39 (1.18–1.64)	<0.001
Max-CIMT(per 0.1 mm increment)	1.58 (1.31–1.91)	<0.001	1.49 (1.24–1.80)	<0.001

Adjusted factors included age, sex, BMI, hypertension, diabetes, smoking, HDL-C, and LDL-C; FRS, framingham risk score; PCE, pooled cohort equations score; CIMT, carotid intima-media thickness; OR, odds ratio; CI, confidence interval.

### 3.7 Incremental predictive value of CIMT for different risk groups

To evaluate the risk reclassification potential of CIMT across different risk categories defined by FRS and PCE, we conducted subgroup analyses (Table 7). The results demonstrated that CIMT had the most significant risk reclassification effect in the intermediate-risk groups (FRS 10%–20% and PCE 5–7.5%). In the FRS intermediate-risk group, after incorporating maximum CIMT, 19.6% (27/138) of participants were reclassified to high risk, while 8.0% (11/138) were reclassified to low risk. Similarly, in the PCE intermediate-risk group, the addition of maximum CIMT resulted in 20.9% (24/115) of participants being reclassified to high risk and 8.7% (10/115) to low risk.

**Table 7:** Effect of CIMT on reclassification of different risk groups.

Risk stratification	*N*	Reclassification after maximum CIMT addition		
		Reclassified to higher risk	Risk stratification unchanged	Reclassified to Lower Risk
FRS Risk stratification				
Low risk (<10%)	99	14 (14.1%)	85 (85.9%)	-
Middle risk (10%–20%)	138	27 (19.6%)	100 (72.5%)	11 (8.0%)
High risk (>20%)	91	-	83 (91.2%)	8 (8.8%)
PCE Risk stratification				
Low risk (<5%)	111	15 (13.5%)	96 (86.5%)	-
Middle risk (5%–7.5%)	115	24 (20.9%)	81 (70.4%)	10 (8.7%)
High risk (>7.5%)	102	-	94 (92.2%)	8 (7.8%)

FRS, framingham risk score; PCE, pooled cohort equations score; CIMT, carotid intima-media thicknessa.

## 4 Discussion

This cross-sectional study evaluated the correlation between carotid intima-media thickness (CIMT) measured by B-mode ultrasound and established cardiovascular risk scoring systems. Our main findings include: (1) CIMT demonstrated significant positive correlations with traditional risk scores such as FRS and PCE; (2) CIMT at the carotid bulb showed stronger correlation with risk scores compared to the common and internal carotid arteries; (3) CIMT was independently associated with high cardiovascular risk categories (as defined by FRS and PCE) after adjusting for traditional risk factors; and (4) CIMT showed the most significant risk reclassification effect in intermediate-risk populations. These results support the value of CIMT as a marker correlating with established cardiovascular risk assessment tools.

We found correlation coefficients of 0.68 and 0.64 between mean CIMT and FRS and PCE, respectively, which align with the results from a meta-analysis conducted by Lorenz et al. (13). Their analysis, which included eight prospective studies, reported correlation coefficients between CIMT and traditional risk scores ranging from 0.60 to 0.70. Polak et al. also reported similar correlations (*r* = 0.67) in the Framingham Offspring Study (24). This moderate-strength correlation suggests that CIMT reflects some, but not all, of the cardiovascular risk expressed by traditional risk factors, thus potentially providing complementary risk information.

Regarding the comparison of CIMT at different carotid artery segments, we observed that the carotid bulb exhibited both the highest CIMT values and the strongest predictive capability. This is consistent with findings from the MESA study, which demonstrated that the carotid bulb CIMT was more predictive of coronary heart disease risk than the common carotid artery (12). Rundek et al. in the Northern Manhattan Study also found that carotid bulb CIMT showed stronger associations with ischemic stroke compared to the common carotid artery (25). This may be because the carotid bulb is a region of hemodynamic force transition and is more susceptible to early atherosclerotic changes (26). However, it should be noted that the consensus statement proposed by Touboul et al. recommends prioritizing common carotid artery CIMT measurements due to their greater reliability and reproducibility (14). Therefore, clinical practice should consider both predictive capability and measurement reliability.

In our multivariate linear regression analysis, we found that age, male gender, hypertension, diabetes, and smoking were significantly positively associated with CIMT, while HDL-C showed a significant negative correlation. Among these factors, age and hypertension emerged as the strongest independent correlates of CIMT. These findings are highly consistent with those of the ARIC study (27). A meta-analysis by Sun et al. further confirmed that CIMT increases by approximately 0.05 mm for every 10-year increase in age (28). Hypertension, as a strong correlate of CIMT, reflects the long-term impact of hemodynamic stress on the vascular wall, as reported by Zanchetti et al. in the ELSA study (29). Additionally, we observed significantly higher CIMT values in men compared to women, which aligns with observations by Sinning et al. in the Gutenberg Health Study (30). These findings reinforce the biological plausibility of CIMT as a comprehensive indicator of atherosclerosis.

A key finding of our study is that CIMT was independently associated with high cardiovascular risk categories (as defined by FRS and PCE) after adjusting for traditional cardiovascular risk factors. It is important to note that these associations were with risk score categories rather than with actual cardiovascular events, as our cross-sectional design did not include prospective follow-up for cardiovascular outcomes.For FRS high risk, each 0.1 mm increase in maximum CIMT was associated with an adjusted OR of 1.58; for PCE high risk, the adjusted OR was 1.49. This result partially aligns with the meta-analysis by Den Ruijter et al., which showed that each 0.1 mm increase in CIMT was associated with a 15% increase in cardiovascular event risk (20). However, Nambi et al. demonstrated that the incremental predictive value of CIMT alone is limited, but significantly improves when combined with plaque information (21). This suggests that we should consider using CIMT and plaque assessment as integrated indicators.

It is crucial to emphasize the distinction between our findings and prospective prediction of cardiovascular events. Our study demonstrates that CIMT correlates with established risk scoring systems (FRS and PCE) that have been validated in prospective studies to predict future cardiovascular events. However, our cross-sectional design does not allow us to directly assess whether CIMT predicts actual cardiovascular events in our population. The associations we observed between CIMT and risk score categories indicate that CIMT reflects the same underlying atherosclerotic burden captured by traditional risk factors, but whether CIMT provides independent prognostic information for future events in our specific population would require prospective follow-up studies. Therefore, our findings should be interpreted as evidence that CIMT is a marker that correlates with established risk assessment tools, rather than as direct evidence of CIMT's predictive value for cardiovascular events.

Our study particularly emphasizes the risk reclassification potential of CIMT in intermediate-risk populations as defined by traditional risk scores. We found that in the FRS intermediate-risk group, after incorporating maximum CIMT, nearly 20% of participants were reclassified to high risk and 8% to low risk. Similarly, in the PCE intermediate-risk group, 21% of participants were reclassified to high risk and 9% to low risk. This is consistent with recommendations from the European Association of Preventive Cardiology, which suggests that CIMT should primarily be used for risk restratification in populations with intermediate risk by traditional risk scores (31). Mathiesen et al. in the Tromsø study also found that CIMT provided a net reclassification improvement (NRI) of up to 23% in intermediate-risk populations (32). These results highlight the potential clinical value of CIMT in guiding primary prevention strategies for intermediate-risk individuals.

Regarding the optimal cutoff value for CIMT, our ROC curve analysis indicated that the optimal cutoff value of maximum CIMT for predicting high FRS risk was 0.93 mm (sensitivity 72.5%, specificity 74.1%). This is very close to the 0.9 mm cutoff value proposed by Bard et al. (33), but higher than the 0.85 mm suggested by Baldassarre et al. (10). This discrepancy likely reflects the heterogeneity of different study populations and variations in measurement methods, emphasizing the importance of establishing population-specific CIMT reference values.

Notably, despite CIMT demonstrating good predictive value, its clinical application still faces several challenges. Research by Costanzo et al. questioned whether adding CIMT to traditional risk models significantly improves predictive accuracy (34). The 2013 American Heart Association guidelines no longer recommend routine use of CIMT for risk assessment (3). However, European guidelines continue to support the use of CIMT in specific populations (1). This controversy reflects ongoing discussions regarding the clinical value of CIMT and suggests the need for larger-scale prospective studies to validate its long-term predictive value.

An important consideration in interpreting our results is the generalizability to broader populations, particularly those without cardiovascular symptoms. Our study population was recruited from a cardiovascular clinic, which may introduce selection bias and limit the applicability of our findings to the general asymptomatic population. Several factors should be considered: First, participants attending a cardiovascular clinic, even those without established cardiovascular disease, may have a higher prevalence of cardiovascular risk factors compared to the general population. In our study, the prevalence of hypertension (46.3%), diabetes (23.8%), and smoking (30.5%) was higher than typically reported in general population studies. This suggests our cohort may represent an intermediate-to-high risk population rather than a truly asymptomatic general population. Second, the relationship between CIMT and cardiovascular risk may differ across populations with varying baseline risk profiles. However, the biological rationale for CIMT as a marker of subclinical atherosclerosis remains valid across different populations. Our findings are consistent with large-scale population-based studies such as the ARIC study and the Rotterdam Study, which included more diverse populations and demonstrated similar correlations between CIMT and cardiovascular outcomes. Third, while our results may not be directly applicable to low-risk asymptomatic individuals, they provide valuable insights for clinical populations with cardiovascular risk factors, which represents a substantial proportion of middle-aged and older adults in clinical practice. The correlation coefficients we observed (*r* = 0.68 with FRS and *r* = 0.64 with PCE) are consistent with findings from population-based studies, suggesting that the fundamental relationship between CIMT and cardiovascular risk is preserved across different study populations. Therefore, while our results should be interpreted with caution regarding their applicability to truly asymptomatic populations, they provide strong evidence for the utility of CIMT in cardiovascular risk assessment among individuals with cardiovascular risk factors or those undergoing cardiovascular screening.

An important limitation of our study is the lack of direct comparison with other established cardiovascular imaging techniques. While we focused exclusively on B-mode ultrasound measurement of CIMT, other imaging modalities have demonstrated significant value in cardiovascular risk assessment. Coronary artery calcium (CAC) scoring, obtained through non-contrast cardiac CT, has shown superior predictive value in several large-scale studies. The Multi-Ethnic Study of Atherosclerosis (MESA) demonstrated that CAC provided incremental predictive value beyond traditional risk factors, with some studies suggesting superior performance compared to CIMT alone. Folsom et al. reported that CAC had a stronger association with cardiovascular events compared to CIMT in the MESA cohort (AUC 0.81 vs. 0.78). However, CAC scoring involves radiation exposure and higher costs, limiting its routine clinical application. Arterial stiffness assessment through pulse wave velocity (PWV) represents another important approach to evaluating cardiovascular risk. The Reference Values for Arterial Stiffness Collaboration demonstrated that PWV independently predicts cardiovascular events and may provide complementary information to CIMT. Ben-Shlomo et al. reported that combining CIMT and PWV measurements improved risk prediction beyond either measurement alone. The ankle-brachial index (ABI) offers a simple, non-invasive assessment of peripheral arterial disease and systemic atherosclerotic burden. While ABI primarily detects lower extremity arterial disease, it serves as a marker of generalized atherosclerosis. Studies have shown that combining CIMT with ABI may enhance overall cardiovascular risk assessment. More recently, coronary CT angiography (CCTA) has emerged as a comprehensive tool for assessing coronary anatomy and plaque characteristics. The SCOT-HEART trial demonstrated that CCTA significantly improved clinical decision-making in patients with suspected coronary disease. However, CCTA involves higher radiation exposure and costs compared to CIMT. Our decision to focus on CIMT was based on its widespread availability, lack of radiation exposure, established evidence base, and cost-effectiveness. However, we acknowledge that comparative studies incorporating multiple imaging modalities would provide more comprehensive insights into the relative strengths and optimal combinations of different approaches. Future research should consider multi-modal imaging studies to determine the most effective and cost-efficient strategies for cardiovascular risk assessment in different populations.

Our study utilized manual B-mode ultrasound measurement for CIMT assessment, which represents the current standard in clinical practice and has been extensively validated in large epidemiological studies. However, we acknowledge that echotracking methods, particularly radiofrequency-based systems, offer superior sensitivity and precision compared to conventional B-mode measurements (35). These advantages may enhance detection of subtle changes in arterial wall thickness and improve cardiovascular risk characterization. Our choice of manual B-mode measurement was based on its widespread clinical availability, well-established standardization protocols (Mannheim Consensus) (14), and comparability with major epidemiological studies (ARIC, MESA, Rotterdam Studies). While the precision limitations of manual measurement may have reduced our ability to detect subtle CIMT differences, particularly in intermediate-risk populations, our findings remain clinically relevant and generalizable to routine clinical practice. Future studies incorporating advanced echotracking technology would be valuable for validating our findings and potentially improving risk assessment precision.

However, this study has several limitations: First and most importantly, as a cross-sectional study, it cannot evaluate the prospective relationship between CIMT and future cardiovascular events. Our findings demonstrate correlation between CIMT and risk score categories, but do not establish that CIMT predicts actual cardiovascular outcomes. The use of risk scores (FRS and PCE) as surrogate endpoints, rather than actual cardiovascular events, is a fundamental limitation that affects the interpretation of our results; Second, the study population was recruited from a single-center cardiovascular clinic, which may limit the generalizability of our findings to the broader general population, particularly truly asymptomatic individuals without cardiovascular risk factors. The higher prevalence of traditional risk factors in our cohort compared to general population studies suggests that our results may be most applicable to intermediate-to-high risk populations rather than low-risk asymptomatic individuals; Third, we did not compare CIMT with other established imaging markers such as coronary artery calcium score, pulse wave velocity, or ankle-brachial index, making it impossible to determine its relative predictive value or evaluate the potential benefits of multi-modal imaging approaches. This represents a significant limitation as comparative imaging studies could provide valuable insights into the optimal combination of imaging techniques for cardiovascular risk assessment; Fourth, we used traditional cardiovascular risk scores (FRS and PCE) as surrogate endpoints rather than actual cardiovascular events; Fifth, while we comprehensively evaluated all individual metabolic components (hypertension, diabetes, lipid parameters, obesity indicators) and their independent associations with CIMT, we did not formally calculate metabolic syndrome prevalence or evaluate it as a categorical diagnostic entity. However, our component-based analysis provides more granular insights into which specific metabolic abnormalities drive the association with CIMT. Recent literature suggests that individual metabolic component analysis may be more informative than categorical metabolic syndrome diagnosis for cardiovascular risk assessment, as different combinations of metabolic abnormalities may confer different risks. Nevertheless, future studies comparing the relative contributions of categorical metabolic syndrome diagnosis vs. individual component analysis in relation to CIMT would provide valuable insights; Finally, the sample size, while adequate for detecting the expected correlations, may limit the precision of subgroup analyses.

Several important research directions emerge from our findings: First and most importantly, prospective longitudinal studies with follow-up for actual cardiovascular events (myocardial infarction, stroke, cardiovascular death) are essential to establish whether CIMT provides independent prognostic information beyond traditional risk scores. Our study demonstrates that CIMT correlates with established risk scoring systems, but only prospective studies can determine whether CIMT independently predicts cardiovascular outcomes. Second, comparative studies incorporating multiple imaging modalities (CAC, PWV, ABI, CCTA) are essential to determine the relative strengths of different approaches and identify optimal combinations for comprehensive cardiovascular risk assessment. Such multi-modal studies could help establish evidence-based algorithms for selecting the most appropriate imaging techniques for different patient populations. Third, cost-effectiveness analyses comparing different imaging strategies would provide valuable guidance for clinical decision-making and healthcare resource allocation. Given the varying costs and accessibility of different imaging modalities, such analyses are crucial for implementation in diverse healthcare settings. Fourth, research focusing on specific patient subgroups (e.g., diabetic patients, patients with chronic kidney disease, different ethnic populations) could help identify populations where CIMT provides the greatest incremental value. Fifth, the role of novel lipid ratio parameters (non-HDL-C/HDL-C, LDL-C/HDL-C, total cholesterol/HDL-C) in improving cardiovascular risk prediction and guiding lipid-lowering therapy warrants further investigation. Our findings, together with recent evidence from Morelli et al. (36), suggest that these ratio parameters may provide superior assessment of atherosclerotic burden compared to individual lipid components, particularly in treated populations. Prospective studies are needed to determine whether incorporating these lipid ratio parameters into risk assessment algorithms can improve prediction of cardiovascular events and whether they can serve as therapeutic targets to guide intensification of lipid-lowering therapy. Finally, investigation of emerging technologies such as 3D ultrasound, contrast-enhanced ultrasound, and artificial intelligence-assisted image analysis could potentially improve the precision and predictive value of carotid ultrasound assessment.

## 5 Conclusions

This study demonstrates that CIMT measured by B-mode ultrasound significantly correlates with traditional cardiovascular risk scores and is independently associated with high cardiovascular risk categories (as defined by FRS and PCE) after adjusting for traditional risk factors among individuals attending cardiovascular clinics. Measurements at the carotid bulb show the strongest correlation with risk scores. CIMT demonstrates significant risk reclassification potential in intermediate-risk populations. It is important to emphasize that our findings are based on cross-sectional associations with risk score categories rather than prospective prediction of actual cardiovascular events. Our results indicate that CIMT is a marker that correlates with established risk assessment tools and may provide complementary information for risk stratification, particularly in individuals with cardiovascular risk factors undergoing cardiovascular screening. However, prospective studies with cardiovascular event outcomes are needed to definitively establish the independent prognostic value of CIMT for predicting future cardiovascular events. However, the lack of comparison with other imaging modalities (such as coronary artery calcium, pulse wave velocity, or ankle-brachial index) in our study represents an important limitation that should be addressed in future research. Multi-modal imaging studies are needed to determine the optimal combination of imaging techniques for comprehensive cardiovascular risk assessment. The generalizability of these results to truly asymptomatic general populations requires further investigation in community-based studies. Future prospective studies incorporating multiple imaging modalities and focusing on actual cardiovascular outcomes across different risk profiles are essential to establish evidence-based guidelines for the clinical application of CIMT and other imaging techniques in cardiovascular risk assessment.

## Data Availability

The original contributions presented in the study are included in the article/Supplementary Material, further inquiries can be directed to the corresponding author.
